# Evaluation of the safety and tolerability of a nutritional Formulation in patients with ANgelman Syndrome (FANS): study protocol for a randomized controlled trial

**DOI:** 10.1186/s13063-019-3996-x

**Published:** 2020-01-09

**Authors:** Donna L. Herber, Edwin J. Weeber, Dominic P. D’Agostino, Jessica Duis

**Affiliations:** 1 Disruptive Nutrition, LLC, 300 West Morgan Street, Suite 1510, Durham, NC 27701 USA; 2 PTC Therapeutics, Inc., 100 Corporate Court, South Plainfield, NJ 07080-2449 USA; 3 Morsani College of Medicine, Department of Molecular Pharmacology and Physiology, University of South Florida, 12901 Bruce B Downs Blvd, Tampa, FL 33612 USA; 4 Institute for Human and Machine Cognition, Ocala, FL USA; 5 Division of Medical Genetics & Genomic Medicine, Department of Pediatrics, Vanderbilt University Medical Center, Nashville, TN 37232-2578 USA

**Keywords:** Ketogenic diet, Ketosis, Angelman syndrome, Placebo-controlled trial, Children, Exogenous ketone, Ketone ester, Ketone supplement

## Abstract

**Background:**

Ketogenic and low-glycemic-index diets are effective in treating drug-resistant seizures in children with Angelman syndrome. Cognition, mobility, sleep, and gastrointestinal health are intrinsically linked to seizure activity and overall quality of life. Ketogenic and low-glycemic diets restrict carbohydrate consumption and stabilize blood glucose levels. The ketogenic diet induces ketosis, a metabolic state where ketone bodies are preferentially used for fuel. The use of exogenous ketones in promoting ketosis in Angelman syndrome has not been previously studied. The study formulation evaluated herein contains the exogenous ketone beta-hydroxybutyrate to rapidly shift the body towards ketosis, resulting in enhanced metabolic efficiency.

**Methods/design:**

This is a 16-week, randomized, double-blind, placebo-controlled, crossover study to assess the safety and tolerability of a nutritional formula containing exogenous ketones. It also examines the potential for exogenous ketones to improve the patient’s nutritional status which can impact the physiologic, symptomatic, and health outcome liabilities of living with Angelman syndrome.

**Discussion:**

This manuscript outlines the rationale for a study designed to be the first to provide data on nutritional approaches for patients with Angelman syndrome using exogenous ketones.

**Trial registration:**

ClinicalTrials.gov, ID: NCT03644693. Registered on 23 August 2018. Last updated on 23 August 2018.

## Background

Angelman syndrome (AS) was first described in 1965 [1], and has a prevalence in the general population of 1:10,000–1:24,000 [2–4]. Loss or reduction of function of the maternal *UBE3A* gene (*ubiquitin protein ligase E3A)* was identified in the late 1990s as the causative mechanism in the majority of cases [5, 6]. Patients with AS universally have global developmental delays including speech impairment and movement disorders [7]. Individuals are typically described as having a happy disposition with frequent laughter. Eighty percent of patients also experience seizures, and all individuals with AS have a disordered electroencephalogram (EEG) [8, 9]. A large proportion of the population also has feeding problems (75%) and other gastrointestinal (GI) complaints [7, 10]. Unfortunately, AS has no cure. Typical treatment protocols include pharmacotherapy for seizures, physical and behavioral therapies, and educational interventions [11]. However, approximately 77% of the AS population experience treatment-resistant seizures [8].

Refractory epilepsy has been treated successfully with specialized dietary approaches, such as the ketogenic (KD), medium-chain-triglyceride (MCT)-based-, and low-glycemic-index diets (LGIT); these diets have also been successful in AS [12–15]. The KD allows the body to shift from carbohydrate-based metabolism to fat-based metabolism, thus stimulating hepatic ketogenesis. Nutritional ketosis is a state where the blood levels of ketone bodies are significantly above baseline, typically > 0.5 mmol/L. The ketone bodies (acetoacetate and beta-hydroxybutyrate (BHB)) are energy substrates used by mitochondria to produce adenosine triphosphate, and can be used as alternative fuels to glucose by the brain, heart, and skeletal muscles.

The high rate of refractory seizures, feeding and GI problems, and severe communication impairments in AS lead to a high unmet medical need. Overwhelming challenges exist to ensuring appropriate diets that serve the complex needs of individuals with AS. Nutritional approaches that promote the body’s use of ketones as an alternative fuel may improve overall nutritional status and allow for a liberalized dietary therapeutic strategy in individuals with AS and, therefore, improve quality of life.

Dietary supplementation with ketogenic ingredients is hypothesized to have an important impact on meeting the nutritional ketosis needs of patients with AS. Ketone supplements can aid in achieving and maintaining ketosis, improve the patient’s overall nutritional health in AS symptom management, and may improve quality of life for patients and their families. This study is designed as the first-ever to evaluate the safety and tolerability of the ketogenic ingredient beta-hydroxybutyrate in a patient population with a high rate of refractory epilepsy. This manuscript describes the rationale, methods, and potential importance of ketone-based nutritional interventions in children with AS.

### Objectives

The primary objective of the trial is to assess the tolerability of a nutritional formulation containing BHB in patients with AS aged 4–11 years on a variety of dietary backgrounds. The secondary objectives include assessment of ketosis when consuming the nutritional formulation and safety of the nutritional formulation in patients with AS. These outcome measures were chosen as this is a vulnerable, nonverbal population with limited ability to communicate difficulties with tolerability and safety concerns. Additionally, the degree of ketosis attained may inform future trials to assess the beneficial impact of patient nutritional status on health outcomes. All measures are assessed at baseline and the end of each 4-week intervention period (placebo versus test formulation).

## Methods/design

### Population and setting

This trial is conducted by Vanderbilt University Medical Center in collaboration with sponsor Disruptive Nutrition, LLC, and the onsite Angelman Syndrome clinic at the Monroe Carell Jr. Children’s Hospital at Vanderbilt is the single study site. The study is funded by the Foundation for Angelman Syndrome Therapeutics (FAST). The research team at Vanderbilt is composed of lead investigator Jessica Duis, MS, MD (pediatric geneticist, Assistant Professor, Division of Medical Genetics and Genomic Medicine, Director, Prader-Willi Syndrome Comprehensive Clinic at Vanderbilt and Director of the Angelman Syndrome Comprehensive Clinic at Vanderbilt), who is responsible for overseeing the trial, including recruitment and taking consent, the majority of clinical assessments of the subjects, and data analysis. Robert Carson, MD, PhD (pediatric neurology and epilepsy specialist, Assistant Professor of Pediatrics at Vanderbilt University), will be the lead neurologist for the study with the responsibility of electroencephalogram (EEG) data interpretation. Fenna Phibbs, MD, MPH (neurology, Associate Professor of Neurology, Vanderbilt Department of Neurology, Vanderbilt University Medical Center) will conduct the mobility tracking. Alexandra Key, MD, PhD (Research Associate Professor of Hearing & Speech Sciences and Psychiatry & Behavioral Sciences; Associate Director, IDDRC Translational Neuroimaging Core C; Director, Vanderbilt Kennedy Center Psychophysiology Lab) will be responsible for collection of the event-related potential (ERP) data and analysis. Patience Ergish, MS, RD, LDN (Clinical Dietician at Vanderbilt University), will collect patient diet information during clinic visits. LeeAnna Melton, RN, BSN, CCRP (Research Nurse Specialist III, Division of Medical Genetics at Vanderbilt University) and Lakin Householder (Clinical Trials Associate II, Vanderbilt University) are the study coordinators and point of contact for families enrolled in the trial. This Vanderbilt research team has primary responsibility for data management and analysis, with coordinated efforts from Disruptive Nutrition’s scientific staff. The Vanderbilt research team meets on a weekly basis.

Additional oversight and auditing of the trial is provided by Donna Herber, PhD, Chief Science Officer of Disruptive Nutrition, LLC with weekly conferences with members of the Vanderbilt research team. In-person meetings are planned quarterly, with data auditing semi-annually. A medical and research advisory committee has been established and consists of independent experts and key opinion leaders. The committee meets semi-annually. The Data Safety Monitoring Board is discussed in a separate section of this protocol under “Data Monitoring.”

Patients are recruited to the Nashville, Tennessee site at Vanderbilt University. Subjects with AS are recruited through the lead investigator’s practice, physician referrals, outreach through social media, and parent support groups such as the Foundation for Angelman Syndrome Therapeutics and the Angelman Syndrome Foundation. Subjects are enrolled with the following inclusion criteria: genetically confirmed diagnosis of Angelman syndrome; age 4–11 years; currently on a LGIT, KD (conventional 4:1 or 3:1, MCT, modified Atkins), or standard diet (regular diet) consistently for at least 3 months; willingness to consume the investigational formulation; and willingness to undergo protocol examinations at home and clinic visits. Subjects are excluded if they: require parenteral nutrition; have major hepatic or renal dysfunction; have a history of diabetes or have diabetes; are significantly underweight with a body mass index < 18.5; participate in other clinical intervention studies within 1 month prior to entry to the study; are unwilling to comply with the protocol requirements; or have contraindications for the use of ketogenic or low-carbohydrate diets which, in the opinion of the investigator, may influence a patient’s ability to participate in the study. Subjects are permitted to continue using all previously prescribed medications, provided the prescriptions have not changed in the 1 month prior to randomization. Parents/guardians of all eligible subjects must sign informed consent prior to study enrollment, following adequate explanation of the aims, methods, objectives, and potential hazards of the trial by the responsible investigator (see Additional file 3).

### Study design

**Fig. 1:**
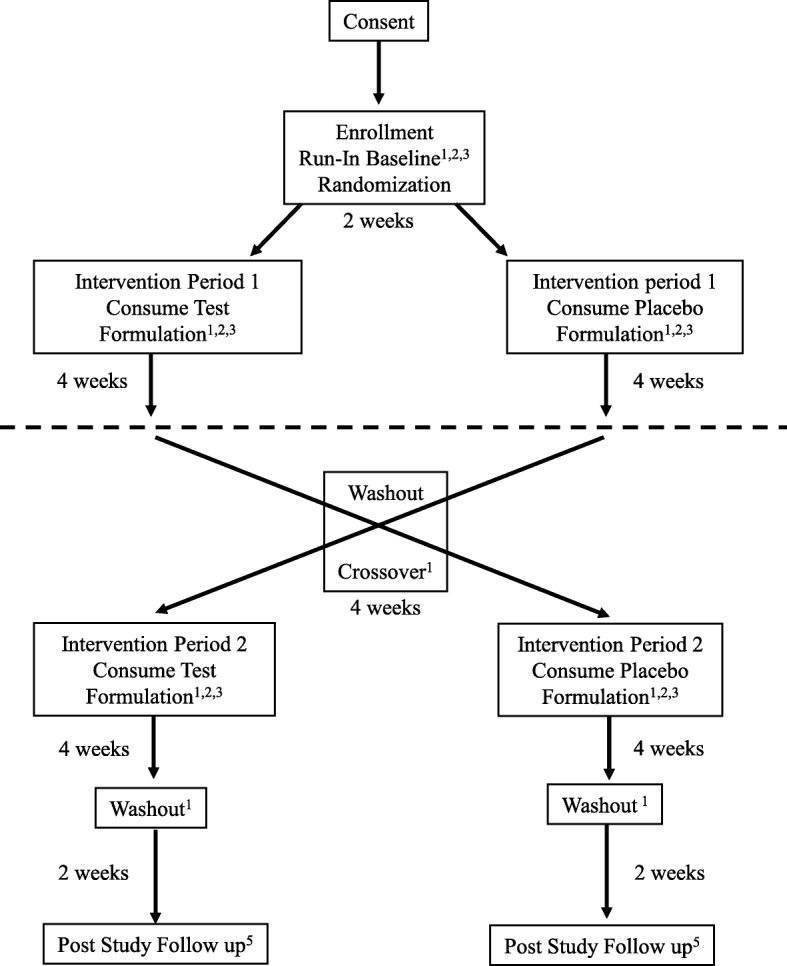
FANS trial design – randomized, placebo-controlled, crossover study. Standard Protocol Items: Recommendations for Interventional Trials (SPIRIT) Figure; schedule of enrollment, interventions, and assessments. *May be conducted by telephone or in person at the clinic site. **An additional sample of blood will be collected during the baseline visit and added to a repository. Samples may be used for studies such as DNA and RNA, proteomics, and metabolomics and may be used for ancillary studies unrelated to the current trial. At the time of publication, no other studies have been planned

This investigation is a 16-week, randomized, crossover, double-blinded, placebo-controlled exploratory study of the safety and tolerability of a nutritional formulation containing *d*, *l*-beta-hydroxybutyrate mineral salts (sodium, calcium, magnesium) (trumacro™, Disruptive Nutrition, LLC, Durham, NC, USA) for use with a KD, a LGIT diet, and standard diet in subjects with AS. It is being conducted in accordance with the Declaration of Helsinki and is approved by the Institutional Review Board (IRB) at Vanderbilt University Medical Center (protocol number DUISJ10212017084407). See Fig. 1 (Standard Protocol Items: Recommendations for Interventional Trials (SPIRIT) Figure: schedule of enrollment, interventions, and assessments), Additional file 1 for the SPIRIT 2013 Checklist (recommended items to address in a clinical trial protocol and related documents), and Additional file 2 for the schematic of the trial design.

Members of the Vanderbilt research team, including the lead investigator and clinical trial coordinators, are responsible for obtaining informed consent (see Additional file 3). Consent is primarily sought through remote interview prior to scheduling the baseline visit in order to reduce travel burden on patients and families. On the consent form, participants will be asked if they agree to the use of their data and banked blood samples should they choose to withdraw from the trial. Participants will also be asked for permission for the research team to share relevant data with people from the institution taking part in the study as well as regulatory agencies where required. After obtaining informed consent from the parent/caregiver, subjects enter a 2-week run-in period. At the beginning of the run-in period the natural history and medical history is collected from the subject’s parent/caregiver. At baseline, a 3-day recall record of the subject’s diet, appetite, GI health, sleep, seizure, mood, and activity level are collected. While one goal of this study is to assess the effects of dietary background on the suitability of the nutritional formulation, there is no requirement for a specific number of patients per diet due to the rarity of the population. Additional study measures, including physiologic, are also collected (Fig. 1).

The study design for this nutritional intervention protocol is a randomized, double-blind, placebo-controlled, crossover study. There are two arms to the study, (1) an investigational formulation period followed by a placebo formulation period, or (2) a placebo formulation period followed by investigational formulation period. Each patient will receive both the investigational formulation and the placebo formulation, with a washout period between each arm. The randomization schedule was created by the Investigational Drug Service Pharmacy using randomization software in a 1:1 ratio with a block size of 4. Participants are enrolled in the next open slot of on the randomization table. The nutritional formulations are coded by the manufacturer lot number and, therefore, the clinical staff is blind to the actual contents of the container (active verses placebo).

Once randomized, subjects start Intervention Period 1, a 4-week period in which they receive nutritional formulation containing BHB or placebo while maintaining the diet noted at entrance into the study. Home monitoring occurs as indicated in Fig. 1. After 4 weeks of blinded intervention, subjects are evaluated in the clinic and then cease consumption of the ketone formulation or placebo for 4-week washout period. Following completion of the washout, subjects enter a second 4-week period in which they receive formulation versus placebo. They will continue the home monitoring throughout the duration of the study. Subsequently, at the completion of the second intervention, they undergo the final scheduled study visit and laboratory sampling. A washout period follows Intervention Period 2, and at approximately week 16 of the study, a follow-up call is made to the family to review symptoms and ensure the patient is not experiencing adverse events during this period. The trial schedule of enrollment, interventions, and assessments is outlined in Fig. 1 (FANS trial schedule of procedures).

### Procedures and interventions

Background information regarding the subject’s natural history is collected using a REDCap© survey (Copyright 2006–2013 Vanderbilt University. All rights reserved) [16]. Body weight and height are measured at each study visit. Serum laboratory analysis is conducted to establish a metabolic baseline: comprehensive metabolic panel, complete blood count, ketones (beta-hydroxybutyrate and acetoacetate), and lipid panel. EEG, ERP and gait analysis using the ProtoKinetics Zeno™ Walkway are performed at each study visit (Fig. 1). The utility of ERP and gait analysis to predict changes in patient function in an Angelman population has not been clearly established, but their appropriateness in similar populations has been detailed elsewhere [17, 18]. Questionnaires completed during the study include the Vineland™-3, Developmental Behavioral Checklist (Pearson Education, Inc.), which has been used previously in studies of patients with AS [19], and the Callier-Azusa Scale to assess areas of motor development, perceptual abilities, daily livingskills, language development, and socialization. Dietary intake, seizure frequency, sleep, and GI health are evaluated throughout the protocol through at-home monitoring. Data collected during home monitoring is recorded on tablet devices provided to the parents/caregivers upon enrollment, preloaded with necessary tracking applications. Families are provided an EarlySense® monitor with a written guidance for home sleep monitoring. External sleep monitors are important in an AS population as wearable devices are typically removed by the patient. The monitors have not been studied with patients with AS; however, their validity has been demonstrated in the general population [20]. Urinary ketones are measured by the patient’s caregivers throughout the protocol, using Ketostix which are a validated measure of urine acetoacetate [21, 22].

Tolerability is determined at the end of each 4-week intervention period. Protocol compliance is monitored during both investigational and placebo intervention periods by recording the amount of formulation consumed. Formulation acceptability is assessed at the end of each intervention period by a parent questionnaire evaluating convenience, taste, and degree of acceptance of the nutritional formulation on a 10-point Likert scale or based on compliance with the study protocol and consumption of the formulation three times daily consistenty during the 4-week interventional period. Similar ranking systems to assess formulation acceptability have been used with other nutritional interventions in epilepsy populations [22–25].

Safety is assessed throughout the entire protocol period through (1) adverse event reporting and (2) assessment of clinical parameters collected throughout the protocol including anthropometrics, blood metabolism profile, dietary intake, seizure frequency, EEG, ERP, mobility, GI function, and sleep habits. In this population, the potential for negative effects of intervention must be closely monitored as patients are nonverbal. Additional file 4 contains details on collecting, assessing, reporting, and managing solicited and spontaneously reported adverse events.

Investigational and placebo formulations are administered with the serving size based on subject body weight. The investigational formulation provides beta-hydroxybutyrate, 2 g carbohydrate, 1 g protein, and 9 g fat, plus minerals, per 100 kcal. The placebo is matched for mineral content only. The formula is given orally as food or beverage three times per day.

Families will be encouraged to continue participation in the trial through weekly telephonic interviews. Data will be collected through three primary interfaces. Background information regarding the patient’s natural history will be collected by the clinical staff using the REDCap© survey. REDCap© is a secure web application that manages the trial database (21 CFR Part 11 and HIPAA compliant). Clinical visits will use either paper or electronic medical records. Data collected during home monitoring will be recorded using a tablet provided to the patient upon enrollment. The tablet is preloaded with the applications necessary to record all data during home monitoring. All data collected, regardless of patient completion status, will be shared with patients upon conclusion of the trial (last patient last visit) and assessment of the data. Additional file 4 contains details on data management and collection procedures.

### Statistical considerations

The primary outcome of the trial is to assess the tolerability of a nutritional formulation containing BHB in patients with AS. Tolerability is demonstrated through patient compliance with the protocol as determined by the amount of formulation consumed as compared to the amount prescribed and is assessed at the end of each intervention period. Premature discontinuation of the nutritional formulation by the family is also deemed as potential intolerability. Tolerability is also assessed at the end of each intervention period through a parental questionnaire. These outcome measures were chosen as this is a vulnerable, nonverbal population with limited ability to communicate and, therefore, determining if the formulation is suitable and well tolerated is an appropriate first step.

The secondary outcomes include assessment of ketosis when consuming the nutritional formulation and safety of the nutritional formulation in patients with AS. Ketosis is evaluated daily through urine testing as well as blood analysis at the end of each intervention period. The degree and timing of ketosis may be clinically relevant to demonstrating better patient nutrition as nutritional ketosis has been related to better patient outcomes as described in the background of this manuscript. Safety is evaluated by changes in motor function, cognitive function, GI tolerance, sleep, and seizures, along with changes in height, weight, and blood metabolic panels as assessed at the end of each intervention period. In addition, adverse event reporting is monitored in real time by the study coordinator.

No studies testing ketone supplementation in patients with AS exist. Therefore, sample-size estimation is based on measures of ketosis. It is assumed from murine pre-clinical investigations, non-AS clinical studies using MCT and/or BHB, and low-carbohydrate dietary interventions, that the minimal change of the secondary outcome, degree of ketosis, from the baseline to the end of the fourth week of the investigational formulation intervention period will be 100% change from baseline (baseline for standard diet is typically < 0.5mmol/L of d-BHB in blood, or < 5 mg/dL acetoacetate in urine). The acetoacetate test used is Ketostix (Bayer Corp. Diagnostics Division, Tarrytown, NY, USA) which exhibits a sensitivity of 78% and a specificity of 96% when compared to a serum standard of 14.4 ml/dL. Considering a 25% drop-out rate, an estimate of 25 subjects need to be screened to achieve 15 completers.

Descriptive statistics, such as mean and standard deviation, are used to describe continuous variables, and frequency for the categorical variables. Means are compared using a paired two-sample *t* test. Statistical significance for all tests is *p* < 0.05. Multiple comparisons can be made including: (1) each subject to serve as their own control, comparing investigational formulation to placebo formulation, as well as to baseline, (2) each dietary background group considered in aggregate, comparing investigational formulation to placebo formulation, as well as to baseline, and (3) all subjects considered in aggregate, comparing investigational formulation to placebo formulation, as well as to baseline.

Any patient who receives the study formulation for any amount of time will be included in the intent-to-treat population for study analyses. Missing data points in this population will be addressed in discussion of any published results. Data from protocol-compliant participants is the defined population for efficacy subset analyses; any participant who received both protocol-required formulations and all required clinical assessments is considered protocol compliant. Such an efficacy subset analysis will be the primary approach for determining whether the trial endpoints are met. Those patients who complete the baseline visit, but do not start Intervention Period 1 and are withdrawn from the study, will be used for natural history reportable data only but will not be included in analyses for determining whether the outcome measures of the trial have been met.

### Discontinuation/withdrawal from study participation

Participation in this study is voluntary. The parent or guardian of a participant may withdraw consent to participate in the study at any time for any reason that they deem necessary. The administration of the study formulation may be discontinued abruptly and does not require any further medical intervention and no alternate therapy will be offered.

### Data monitoring

In order to ensure the safety of the study participants, members of a Data Safety Monitoring Board (DSMB) have been appointed by the principal investigator and Vanderbilt University. The board consist of a chairperson, biostatistician, and three independent reviewers with expertise in the research field. Each member of the board meets these minimum qualifications including: (1) expertise in the field, (2) experience in the conduct of clinical trials and statistical knowledge, (3) independence from the direct management of the clinical trial, and (4) no conflict of interest. The chairperson is responsible for overseeing the meetings, developing the agenda, summarizing the meeting, and is the contact person for the DSMB. The DSMB meets three to four times per year, such schedule to be determined by the chairperson. Additional details on the charter of the DSMB can be had by contacting the Vanderbilt University Medical Center Human Research Protections Program at www.vumc.org/irb.

### Ethical considerations

This study is to be conducted according to US and international standards of Good Clinical Practice (FDA Title 21 part 312 and International Conference on Harmonization guidelines), applicable government regulations and institutional research policies and procedures. This protocol and any amendments will be submitted to the Vanderbilt University IRB in agreement with local legal prescriptions, for formal approval of the study conduct. The DSMB will work in conjunction with the principal investigator and IRB to determine whether a protocol amendment warrants additional patient consent or other communication to the enrolled patients and their families. The decision of the IRB concerning the conduct of the study will be made in writing to the investigator before commencement of this study. Protection of participant confidentiality is described further in Additional file 4.

Participants may be able to claim compensation for injury caused by participation in this clinical trial. Participants who sustain injury and wish to make a claim for compensation should do so in writing in the first instance to the principal investigator, who will pass the claim to the sponsor’s insurers, via the sponsor’s office.

## Discussion

Angelman syndrome is characterized by refractory epilepsy. In fact, the EEGs of individuals with AS are abnormal when compared to neurotypical controls even in the absence of seizures [26]. Fasting has been recognized as a potential treatment for seizures since ancient times, and dietary intervention that mimicked fasting was introduced in the early 1920s [27, 28]. These diets place the body in a state of nutritional metabolic ketosis. Using nutritional ketosis as a model, many dietary interventions have been studied in both animal models and in human clinical trials for a variety of neurologic disorders.

For children with refractory epilepsy, dietary interventions leading to nutritional ketosis are efficacious and safe. A modified Atkin’s diet, prospectively studied in children aged 3–18 years, demonstrated that moderate urinary ketosis developed within 4 days with significant improvement in seizure frequency at 6 months [29]. In AS, case reports also demonstrate efficacy [30]. For a 5-year-old girl with uncontrollable daily seizures resistant to triple-anticonvulsant therapy, a KD proved to be effective in diminishing seizures from the first week of initiation. An EEG with no epileptic activity confirmed the patient’s improvement along with improvement in sleep and hyperactivity. In a separate study in patients with AS, 4 months of LGIT resulted in decreased seizure frequency with correlative improvement post-trial EEG, with evidence of developmental progress. The diet was well tolerated and five of six subjects remained on the LGIT after completion of the trial [14]. A retrospective medical record review of 23 subjects with AS who utilized LGIT suggested that 22% of subjects maintained complete seizure freedom, 43% maintained seizure freedom except in the setting of illness or non-convulsive status epilepticus, and 30% had a decrease in seizure frequency [13].

The literature suggests a safe transition from glucose to ketone bodies as fuel. A large study of the use of the LGIT diet in pediatric epilepsy revealed limited side effects and decreased seizure frequency [31]. Studies of the MCT diet revealed GI complaints as the greatest tolerability issue [32]. Acidosis has also been reported. The risk of nutritional deficiency in children on restrictive dietary treatments and a lack of KD-specific supplements raised concerns about micronutrient status. Vitamins A and E, zinc, selenium, calcium, and magnesium levels have been measured in children with intractable epilepsy treated with the KD [33]. The researchers suggested that changes in plasma vitamins A and E and the decline in magnesium status after 12 months may be suboptimal and require supplemental approaches to the KD.

While clinical evidence suggests that nutritional ketosis through diet is effective, well tolerated and safe in seizure control; little clinical evidence is available regarding the consumption of exogenous ketones as part of nutritional support to improve a patient’s overall health, supporting dietary and pharmacologic management of seizures and other symptoms. Such an approach is supported by animal models. Transgenic mice, altered at the *E6-AP ubiquitin ligase* gene which is known to be the causative factor in AS, resemble human AS with motor dysfunction, inducible seizures, and a context-dependent learning deficits [5, 6, 34]. These mice were fed an exogenous ketone (R, S-1,3-butanediol acetoacetate diester) ad libitum for 8 weeks, mixed into the standard rodent chow at 10% weight/weight. The animals were in nutritional ketosis and demonstrated improved motor coordination, learning and memory, and synaptic plasticity [35]. Exogenous ketone supplements (ketone ester 2.5 g/kg/day; BHB salt + MCT: 2.5 g/kg/day) administered orally for 7 days to genetic absence-like epilepsy Wistar Albino Glaxo/Rijswijk (WAG/Rij) rats that have modulated absence epileptic activity showed significantly decreased number of spike-wave discharges. These improvements returned to baseline levels on the first day without ketone supplementation [36].

The animal evidence supporting ketone supplementation and the clinical evidence for KDs in models of AS and seizure control, respectively, suggest that dietary interventions including exogenous ketones may be an important development in clinical management strategies. The current study is designed as an initial evaluation of ketogenic formulations used with a variety of dietary backgrounds. It represents a unique opportunity to study the potential benefits of nutritional intervention in a pediatric population (ages 4–11 years) with AS. The knowledge gained from the study may guide further evaluations in the use of exogenous ketones for nutritional strategies in the dietary management of patients AS.

It is known in the AS population that there is a large placebo effect. Our study also offers a unique design in which the use of objective measures is prioritized. We hope that this will yield measures that benefit future trials in the Angelman community in addition to a clearer understanding of the overall effectiveness and mechanism of action of BHB for inducing ketosis. The ultimate goal will be to positively impact nutritional status, which should enhance the ability of the patient to overcome the symptoms of AS such as seizures, cognition and learning, gait/mobility, and/or sleep disturbances.

## Trial status

ClinicalTrials.gov, ID: NCT03644693. Registered on 23 August 2018. Last Updated on 23 August 2018; recruitment start date November 2018; approximate completion date December 2019.

## Supplementary information

**Additional file 1.** Standard Protocol Items: Recommendations for Interventional Trials (SPIRIT) 2013 Checklist.

**Additional file 2.** Schematic of trial design.

**Additional file 3.** Patient consent document.

**Additional file 4.** Additional protocol details.

## Data Availability

Data sharing is not applicable to this article as no datasets were generated or analyzed during the current study. The principal investigator and clinical team will have access to the final dataset. The research team at Disruptive Nutrition will only have access to de-identified data.

## Abbreviations

AS: Angelman syndrome
BHB: Beta-hydroxybutyrate
EEG: Electroencephalogram
ERP: Event-related potential
GI: Gastrointestinal
IRB: Institutional Review Board
KD: Ketogenic diet
LGIT: Low-glycemic-index therapy
MCT: Medium-chain triglycerides
UBE3A: *Ubiquitin protein ligase E3A*
