# Supplementary material for: Evaluation of the safety and tolerability of a nutritional Formulation in patients with ANgelman Syndrome (FANS): study protocol for a randomized controlled trial
Source: Trials. 2020 Jan 9;21:60. doi: 10.1186/s13063-019-3996-x (PMC6953273; doi:10.1186/s13063-019-3996-x)
Supplement: Supplementary file 2 — Additional file 2. Schematic of trial design. [file 13063_2019_3996_MOESM2_ESM.pptx]

## Slide 1
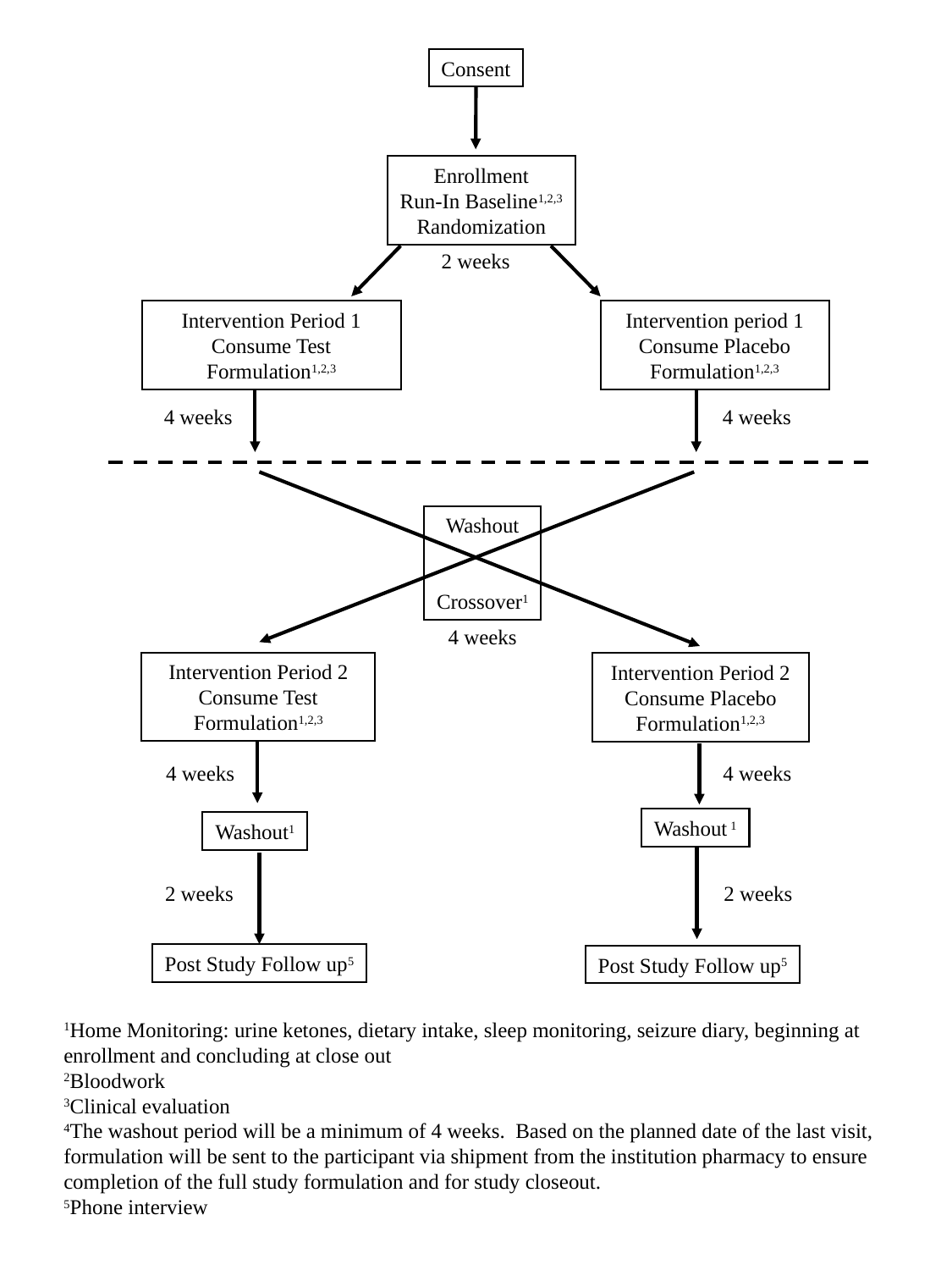

Consent
Enrollment
Run-In Baseline1,2,3
Randomization
2 weeks
Intervention Period 1
Consume Test Formulation1,2,3
Intervention period 1
Consume Placebo Formulation1,2,3
4 weeks
4 weeks
Washout
Crossover1
4 weeks
Intervention Period 2
Consume Test Formulation1,2,3
Intervention Period 2
Consume Placebo Formulation1,2,3
4 weeks
4 weeks
Washout 1
Washout1
2 weeks
2 weeks
Post Study Follow up5
Post Study Follow up5
1Home Monitoring: urine ketones, dietary intake, sleep monitoring, seizure diary, beginning at enrollment and concluding at close out
2Bloodwork
3Clinical evaluation
4The washout period will be a minimum of 4 weeks. Based on the planned date of the last visit, formulation will be sent to the participant via shipment from the institution pharmacy to ensure completion of the full study formulation and for study closeout.
5Phone interview
