# Supplementary material for: Evaluation of the safety and tolerability of a nutritional Formulation in patients with ANgelman Syndrome (FANS): study protocol for a randomized controlled trial
Source: Trials. 2020 Jan 9;21:60. doi: 10.1186/s13063-019-3996-x (PMC6953273; doi:10.1186/s13063-019-3996-x)
Supplement: Supplementary file 3 — Additional file 3. Patient consent document. [file 13063_2019_3996_MOESM3_ESM.pdf]

Study Type and Performance Site Information

Type of study:

[x] **Standard or Expedited**
[ ] Exempt
[ ] Grant Review/Umbrella Review for funds release
[ ] Comparative Effectiveness Research
[ ] Non-Human Subject Determination
[ ] Quality Improvement/Non-Research Determination
[ ] Request review by another IRB
[ ] Coordinating Center ONLY

Please indicate which Committee is most appropriate to review your project:

[ ] Social and Behavioral Sciences
[x] **Health Sciences**

Are there sites in this study in which the PI is responsible other than Vanderbilt University or Vanderbilt University Medical Center, including all VUMC clinics and hospitals or are you requesting the VUMC IRB be the IRB for other sites?

[x] **Yes**
[ ] No

Are there any international sites involved?

[x] **Yes**
[ ] No

Are the sites "Engaged in the Research"?

[x] **yes**
[ ] no
[ ] both engaged sites and not engaged sties

Are you requesting the VUMC IRB to be the IRB of Record for any site?

[ ] Yes
[x] **No**

Is this project cancer-related?

[ ] Yes
[x] **No**

Study Purpose and Description

Provide a brief abstract of the study in lay language. The IRB Committees are comprised of scientists with varied backgrounds, non-scientists, and community members.

Studies indicate treatment success of low glycemic treatment (LGIT) and ketogenic diets for seizures in Angelman syndrome. Our goal is to study the safety and tolerability of a fat-based nutritional intervention in children with Angelman syndrome on LGIT, ketogenic or standard diets. Initial enrollment of the study will be open to 4-11 year old children with Angelman syndrome. We will first track baseline status for 2 weeks. Individuals will come in for a visit and be provided an electronic device (tablet) with preloaded applications to track seizures, food intake, gastrointestinal symptoms, sleep habits, and baseline ketone production. At the initial clinical visit, they will have clinical studies including a baseline laboratory assessment, Vineland-3 assessment, electroencephalogram (EEG), mobility assessment with the ProtoKinetics Zeno walkway, and an evoked-related potential (ERP). The study will be a double-blinded placebo controlled crossover study in which initially children will be assigned to either placebo or the nutritional intervention arms of the study each for a 4 week period with a 4 week wash-out period between crossover of subjects. During the duration of the study, individuals will continue with recording data in the provided applications as noted in the baseline period. There will be three clinical visits associated with the study that will include clinical intake, physical examination, laboratory assessment, administration of the Vineland-3, EEG, gait-tacking analysis, and ERP to study the safety and tolerability of the intervention to ensure nonverbal children do not have safety concerns that they are unable to articulate. These studies will detect hypoglycemia, changes in electrolytes, seizures, and changes in cognition, adaptive function or mobility that could be affected by the nutritional intervention.

Expected duration of the study.

1 year

The IRB needs to understand how this study adds to the knowledge on this topic in order to be able to judge the risks and benefits to the research participants.

Angelman syndrome (AS) is a rare disorder present in approximately 1/12,000-24,000 individuals. Characteristics of the disorder include seizures often refractory to standard therapies, developmental delays including no development of language, severe intellectual disabilities, behavioral problems, gait abnormalities such as ataxia and delayed walking followed by decreased mobility as individuals get older. To date, there is no cure nor are there any targeted therapies. Nutritional manipulation of diet to decrease carbohydrate content by use of low glycemic index therapy (LGIT) or ketogenic diets is beneficial therapy for seizures and may help behavioral problems in AS. Studies in animal models of AS suggest use of nutritional supplements to increase circulating ketones as an alternate fuel for brain metabolism reverses phenotypical features of AS in these models; for example, use of a ketone ester in an AS mouse model consistently reversed motor deficits in the mice. To date, no studies show whether boosting the transition to alternate fuels in the body through nutritional supplementation with a ketone body is safe and tolerable to patients with AS. Studies using similar products suggest safety including of products to help quickly induce ketosis in the transition to a ketogenic diet such as with ketocal and this is now a standard of care in individuals transitioning to this diet. In this phase IIa study we aim to study the safety and tolerability of nutritional supplementation in children with AS with home monitoring, studies of gait and mobility, measures of adaptive functioning, seizure activity and cognition.

Date of IRB Approval: 12/20/2018
Institutional Review Board
VANDERBILT

Research, Activities, Procedures, and Schedule of Events for Study Participants

Please check all that apply to your study and describe each below.

[**x**] **Behavioral Observation**
[**x**] **Randomization**
[**x**] **Blinding**
[**x**] **Surveys, Interviews, Questionnaires**
[ ] Document and Artifact Collection
[ ] Deception, Withholding or Postponing Medications/Treatments, or Imposing other Restrictions
[**x**] **Audio/Video Recording**
[ ] Sham Procedure
[**x**] **Specimen/Data Collection and/or Storage**

DATA COLLECTION, STORAGE OF DATA/SPECIMENS, AND/OR ISSUES OF CONFIDENTIALITY - Describe the procedures that will be utilized to protect the privacy of the research participant. Include who will have access to the research information (for example, video/audio recordings, discovering information about the participant that could be harmful if released such as mental illness, genetic information, sexual preference, drug abuse, etc.) and where it will be stored.

Each participant will be assigned a unique study number by the study PI or coordinator to protect the participant's personal health information (PHI). Personal information will only be used by the study coordinator or PI in the clinical setting. A master database containing participants names and unique study number will be stored as a secured file with password protection on a VUMC server. All data collected from the study participants will be de-identified prior to leaving the clinical setting by entering specific data points into an electronic data collection program according to study number. This process will protect the participant's personally identifiable information. All data entered into the tablet will be appropriately de-identified and be tracked by the study number.

Data management will be the responsibility of the study coordinator. He/she will maintain the electronic study database, study worksheets, and informed consent. Although any document leaving the clinic setting is to be de-identified, all study data will be stored securely in electronic format on the Vanderbilt University secure server. Despite de-identification, only HIPPA trained study personal will have access to the study data base.

Date of IRB Approval: 12/20/2018
Institutional Review Board
VANDERBILT

Describe how the confidentiality of participants' data will be assured. Include a description of any issues specific to the study that might increase the risk of breach of confidentiality. Describe how codes will be generated if codes are used to protect identities, and who will have access to such codes. If a certificate of confidentiality will be provided, include the name of the person holding the certificate. Describe the final disposition of research data when the study is concluded (e.g., will information be destroyed, will the PI maintain the information indefinitely, etc.).

This study will be performed according to all Vanderbilt University Medical Center privacy policies as well as federal, state and local regulation. In accordance with HIPPA regulations all "individually identifiable health information" will be removed (de-identified) from any source documents before leaving the clinical site. Participants enrolled in this study will be assigned a study number which will be used to identify a particular data source. A master list will be made containing patient name, date of birth and study number and stored securely on a password protected server accessible to the study PI and coordinator. De-identification of study data will be achieved by reviewing source documents while in the clinical setting and transferring specific data points into a secure data collection software program according to a participants study number. Data will be stored on a Vanderbilt University Medical Center secure server. Source documents will be stored securely within an password protected server. Codes will be randomly generated with no relationship to the study participants PHI. Codes will only be accessible by the study PI and coordinator. However, this will only be to ensure appropriate identification of study subjects for the longitudinal collection of data and once the subject leaves the clinical site all data will be de-identified and only accessible by the assigned study number.

With respect to the tablets, the name entered will consist solely of the subject's study ID. No PHI will be utilized on the data available directly on the tablets or the related applications. Tablets will be appropriately encrypted for official use the study.

At the completion of the study, study groups will be unveiled and data analysis will be conducted by the PI and study team. Data will remain de-identidied. The PI will maintain the information indefinitely.

BEHAVIORAL OBSERVATION - Describe the focus, duration, and number of observations and specify how the observations will be recorded.

Behavioral observation in this study will be conducted on three visits that include baseline, after intervention period one and at the conclusion of the second interventional period.
Electroencephalograms (EEG) will last for approximately 1 hour. This will be collected using standard procedures including use of electrodes placed on the child's head for recording of activity.
We will also study cognition with use of evoked potentials and this will also include placement of a cap on the child's head while they view pictures on a screen. This will take approximately 1 hour.
We will observe participants walking on the Zeno Walkway to track gait for approximately 10 minutes. This will include walking in both directions two times. The total time for this segment will be one hour.
We will request that families complete the Vineland-3 through an online tool provided by the publishing company, Pearson. This will be completed after consent in obtained. The first survey will be completed at the initial study visit. Subsequent questionnaires will be done the day of the study visit or the day before depending on the family preference. It will be completed in conjunction with each visit.
Data collection will be recorded by these devices for analysis. There may be use of video during recording to correlate to analysis for example during EEG screening for later correlation with clinical seizure activity.
Sleep will be monitored non-invasively at home using an EarlySense sleep monitor system. This is non-invasive and requires no input from the participant. Other behaviors monitored through applications provided on an tablet will include diaries recording dietary intake, seizures and other information such as GI symptoms. We will also provide links to RedCap surveys to record this information.

RANDOMIZATION - Describe the randomization process (who will randomize, how will randomization be determined, etc.)

The study PI and staff will be blinded to the randomization. Randomization will be performed by the manufacturer of the formulation. It will completed randomly by automatic generation for each participant.

BLINDING - Describe who will be blinded and if/when research results or previously blinded treatment assignments will be made available to participants. Include the provisions for breaking the blind (e.g., emergency situations, participant's request, etc.).

The nutritional formulations (investigational and placebo) will be manufactured by Valentine Enterprises,(VEI) a cGMP certified contract manufacturer located in Lawrenceville, Georgia, USA. The formulations will be tested for potency, microbial contamination, and heavy metals prior to release for use in the study. The dry powder formulations are stable at room temperature for up to one year. VEI will assign a unique identifier to each lot produced, effectively blinding the material from designation as either placebo or investigational formulation. This identifier will be printed on a label and placed on the lids of the bottle. The study team, parents and participants will be blinded to the intervention versus placebo assignment.
The final packaged formulation will be sent directly to the pharmacy at VUMC. The material is stored at room temperature. Scoops will be provided to the pharmacy by VEI in several sizes in order to allow for the correct serving size according to the patient's body weight.

Breaking the blind will occur only in the event of severe adverse reactions linked to the intervention versus placebo formulations to ensure appropriate monitoring and response to these reactions. It will only be completed if there are safety concerns. If participant's request unblinding, it will not be granted unless directly related to the participants safety.

SURVEYS, INTERVIEWS, AND QUESTIONNAIRES - If surveys, interviews or questionnaires will be used as part of this study, indicate who will conduct the survey, interview or questionnaire and his/her qualifications. In addition, describe the setting and mode of administering the instrument (e.g., by telephone, one-on-one, group, etc.) and attach a copy of the instrument.

Participants will be provided with a link to a RedCap survey for initial intake of clinical data and questionnaires which will include a sleep scale, rating of gastrointestinal symptoms and general clinical data including past medical history and current clinical concerns. This will then be reviewed at the initial visit with the study PI and coordinator and may require additional data entry by the study team. Interviews will be conducted by the study PI and/or the study coordinator or nurse practitioners. It will be done during the clinical visits in the clinical research center.

Additionally, we will have families complete the Vineland-3. The Vineland Adaptive Behavior Scales, Third Edition (Vineland-3) is an individually-administered measure of adaptive behavior that is widely used to assess individuals with intellectual, developmental, and other disabilities. We will ask families to complete the Parent/Caregiver form to assess communication, daily living skills, socialization, motor skills and maladaptive behavior. This will include up to 381 items on the comprehensive-level and 120 items on the domain-level.

Date of IRB Approval: 12/20/2018
Institutional Review Board
VANDERBILT

AUDIO/VIDEO RECORDING - Describe how the audio/video recordings will be stored, as well as how they will be disposed of when this research is complete. Describe how the participant's confidentiality will be maintained.

Audio/video recording may be used to ensure complete collection of data and ability to review information. For example, video may be used during the EEG and this video may be used to look for a clinical correlation of seizures should that be noted upon interpretation of the EEG. It could be used during the ERP record and gait mobility as well primarily to correlate data and findings or ensure appropriate and consistent scoring is completed. Videos will be de-identified and the examiner will be asked to use the assigned study number. Videos will be discarded once the scoring and data collected has been completed.

SPECIMEN COLLECTION - Describe all procedures used for specimen collection.

Standard venipuncture will be used for collection of blood samples. Blood samples will be collected for complete blood count, comprehensive metabolic panel, lipid panel, ketone bodies, and an extra lavender top tube for research purposes. This will include collection to 2 light green tubes and a single lavender top tube. The collected specimens will be sent to the laboratory at Vanderbilt for analysis. The lavender tope tube will require immediate processing by the nursing staff in the CRC. It will be spun down and the plasma separated for future study of metabolites. The cellular material will be saved for future research purposes including extraction of RNA and DNA. A total of 5-8 mL will be collected at each visit.

Urine will be collected at home either by use of a cotton ball if the participant is diapered or by having the child urinate in a specimen container provided to the family (either a plastic container that fits the entirety of the toilet bowl or a urinal per each family's preference).

Will you be performing a blood draw(s)?

[**x**] **Yes**
[ ] No

Indicate ml over unit of time.

~5-8 mL per study visit over a 16 week period of time (3 total visits)

Will specimens be obtained for genetic testing?

[**x**] **Yes**
[ ] No

Will the PI create a repository at VU/VUMC with any of the specimens and/or data for future use?

[**x**] **Yes**
[ ] No

Specify the types of tissue or data to be banked. Describe the source of the data or specimens and the tools that will be utilized in the collection and storage of research information.

Specimens will include stored plasma and leukocytes from the spun down specimens collected in the CRC. These will be for future use including study of metabolites in the plasma and extraction of RNA/DNA from the cellular material, or for transformation into iPSC-derived neurons. Data stored will include all information from the applications used during the study including seizures, sleep, GI symptoms, and diet. Additional information will include the laboratory data, Vineland-3 assessment, EEG, event-related potentials (ERP), and data from gait-tracking studies. These will all be stored with a password protected, de-identified database on a secure Vanderbilt server.

Tools used include the Vineland-3, EEG, ERP, Zeno Walkway, and the EarlySense sleep monitor. We will also use an tablet for the collection of data during the study period. Additional tools will include the venipucture material and lavender top tube. It will require the appropriate procedure for extraction of RNA/DNA using standard kits. Storage will be in the Division of Medical Genetic and Genomic Medicine in a -80 freezer.

Are the specimens/data identified/coded?

[**x**] **Yes**
[ ] No

Describe the procedures that will be used to de-identify specimens/data, if applicable.

During the clinic visit, labels will be printed with the appropriate subject code and this will be placed on research specimens. PHI will not appear on specimens collected for research purposes.

Describe the management of the Repository including: • The identity of the person responsible for maintaining the Repository and the identity of any others involved with stripping identifiers, coding or distributing samples/data; • What will happen to specimens if the PI leaves the institution; and • What will happen if an individual decides to withdraw consent.

The PI of the study (Dr. Jessica Duis) will store the materials in the -80 freezers within the laboratories located in the Division of Medical Genetics & Genomic Medicine in Medical Center North. The study coordinator and/ or nursing staff will be involved in appropriate coding and ensuring samples are appropriately labeled prior to placement in storage. They will also be responsible for data management which will be stored on a secure server and password-protected RedCap database.

If an individual withdraws consent, the specimens will immediately be destroyed in the appropriate manner.

Describe the storage facilities and tracking system to be used, including how the specimens/data are received, accessed and released.

Storage will be in a -80 freezer within the laboratories located in the Division of Medical Genetics and Genomic Medicine in Medical Center North. The freezer is locked as well as the door to enter the laboratory and there will be limited access to the specimens which will be managed by the study PI and coordinator. Specimens will be logged into the freezer through a tracking system that includes details of placement and coding. The database will be located on a secure server with password protection only accessible by the PI and study coordinator. Upon access to the specimens this will be recorded in a detailed log maintained by the study coordinator. If release of the specimens will only be granted by the study PI and will only be granted if covered within the approved study.

Date of IRB Approval: 12/20/2018 Institutional Review Board
VANDERBILT

Please indicate all procedures and activities performed for research purposes only and the frequency at which they occur in the study (e.g., skin biopsy, 3 times).

**Procedure/Activity**	**Frequency**
Venipuncture	3 times (baseline, intervention 1 visit and Interventivisiton 2
EEG	3 times (baseline, intervention 1 visit and Interventivisiton 2
ERP	3 times (baseline, intervention 1 visit and Interventivisiton 2
Zeno walkway	3 times (baseline, intervention 1 visit and Interventivisiton 2
Sleep monitoring device	every night used for home monitoring during the study
Check urine ketones at home	daily
Anthropometrics	3 times (baseline, intervention 1 visit and Interventivisiton 2
Record Dietary Intake Daily	daily
Record Seizures Daily	daily
EarlySense Nightly Sleep Monitoring	daily
GI health diary recorded daily	daily
Formulation preparation and intake recorded daily	daily
Rate convenience, taste, and acceptability	daily
Telephone Contact	weekly by study coordinator
Concomitant Medication	3 times(every clinic visit) and every phone call (weekly)
Adverse Events recorded	2 times (every clinic visit except the baseline and every phone call (weekly)
Vineland-3	3 times (baseline, intervention 1 visit and intervention 2 visit)

If all of your study is minimal risk, please indicate the categories that it fits 45 CFR 46.110 or 21 CFR 56.110:

[**x**] **N/A: Study is greater than minimal risk or Standard**
[ ] (F)(1) Drugs or devices where no IND/IDE is required
[ ] (F)(2) Collection of blood by stick or venipuncture
[ ] (F)(3) Prospective collection of specimens by non invasive means
[ ] (F)(4) Collection of noninvasive data through routine clinical practice
[ ] (F)(5) Research on materials that have been collected for non research
[ ] (F)(6) Collection of data from voice, video, digital or image recordings
[ ] (F)(7) Research on individual or group characteristics (surveys)

Data and Safety

Describe how the risks to participants are minimized (e.g., screening to assure appropriate selection of participants, identify standard of care procedures, sound research design, safety monitoring and reporting).

The risks to participants are minimized by screening and approaching only the patients that qualify for the study. The PI and study staff are experienced in reviewing and obtaining consent. The study team members are trained in HIPAA privacy regulations and other applicable privacy policies at VUMC. The study team will be monitored by the sponsor on a regular basis to ensure compliance to the protocol.

All participants at our site will be reported to the sponsor and to the Vanderbilt IRB. We will thoroughly screen individuals prior to participation to ensure there is no history of complications to transition to the ketogenic diet, history of hypoglycemia, or recent worsening of disease, among other factors. Standard care precautions when applicable will to be used. For example, we will take the standard precautions during EEG procedures and venipuncture. In the case of the latter this will decrease the stress to the participant and also the possible complications including infection. Our research design has many different ways in which we can capture adverse effects including home monitoring with tablet applications provided to the families to track seizures, diet and intake, and sleep. RedCap surveys will also be used to tracking GI symptoms during the study through links provided to the participants. These will be reviewed in real-time to ensure these are followed up in a timely manner.

Vanderbilt will serve as a coordinating site for international studies in Australia (2 sites). These sites will require their own IRB approval to protect the participants at those particular sites.

Describe how the risks to participants are reasonable in relation to anticipated benefits (e.g., includes benefits to the individual as well as to human kind, indicate how the risks are justified in this population).

The risks to the individual is the risk of standard venipuncture, risk of consumption of the intervention and placebo and the burden of home monitoring and recoding data. The benefits of the study are to define the safety and tolerability of a nutritional intervention that could be utilized to shift the balance of energy in the body to ketones from glucose. We believe the potential benefit of this intervention could be huge given the mouse data in an AS model that showed reversal of the motor deficits common also in AS individuals. Data also suggests induction of ketosis as occurs in the ketogenic diet and in the low glycemic index therapy improves refractory seizures in AS. Therefore this formulation could be a used in combination with AED therapy and dietary intervention as a nutritional supplement to help improve seizures, gait and mobility concerns. To date, these are aspects of AS for which we do not have treatments. These are also often debilitating concerns that lead to poor quality of life, increased caregiver burden, and possibly death (e.g. refractory seizures). This data will help the individuals who participate in the study and the entire patient population in that these are all characteristics of AS that are common.

Is there a data safety monitor or board/committee to review this study for safety and adherence to the study protocol?

[**x**] **Yes**
[ ] No

Describe the composition of the committee and their qualifications.

The committee will include independent experts and key opinion leaders in fields, which may include but is not limited to parent and scientists knowledgeable about Angelman syndrome, a dietician knowledgeable about the ketogenic diet and induction of ketosis as well as the potential side effects, manufacturers, and scientists with prior experience in nutrition therapies.

There will be a unique committee for the sites in Australia.

Provide a general description of the data and safety monitoring plan.

The committee has already participated in review of the protocol the study PI and will continue to act as an advisory body and will monitor the safety and tolerability of the intervention. The committee will make recommendations for stopping or continuing the study on a subject level per the pre-specified stopping criteria.

Data will be reviewed weekly at the coordinating center (VUMC) for the international sites. They will have their own principal investigators to ensure the safety of subjects enrolled in the study. They will be responsible on a daily basis for reporting any adverse events to the VUMC coordinating site and making the appropriate medical decisions for the participant.

Describe plans for monitoring the progress of trials and the safety of participants (e.g., timing of DSM reviews and reports, planned interim analysis, etc.).

The committee will continue to advise the PI of the study. Their primary role will be to protect the safety of the subjects and to make recommendations concerning continuation of the formulation based on the available clinical data, pharmacokinetics, and additional data. The committee will remain in touch with the PI at regular intervals during the study period. A formal meeting may occur in person at Angelman syndrome meetings, which occur on a national basis every 6 months.

The study will include subjects in three categories at baseline entry into the study. These include standard, low glycemic index therapy (LGIT) and ketogenic diets. We will monitor each groups production of ketones, comprehensive metabolic panel, lipid panels during the duration of the trial to check for signs of any intolerance of the formulation. On the day or two after each visit, the PI will review data and ensure individuals have no signs of abnormalities. On a monthly basis the study PI and study team will discuss the progress of participants in the trial to ensure safety and tolerability.

Site progress of the international sites will be through weekly data review by the study PI and team at VUMC. Direct review of the safety of participants will be per IRB oversight at the specific Australian sites.

Date of IRB Approval: 12/20/2018
Institutional Review Board
VANDERBILT

Describe plans for assuring compliance with requirements regarding the reporting of adverse events (AEs), including plans for reporting of AEs to the IRB and appropriate regulatory agencies.

The study AE reporting period is as follows: After informed consent is obtained and the first administration of study formulation, all non-serious AEs and SAEs reporting period begins and continues until 4 weeks following either the last administration of study formulation or the early termination visit, whichever is longer. Each SAE must also be reported in the CRF. The study PI will follow all unresolved SAEs until the events are resolved or stabilized, or it has been determined that the study treatment or participation is not the cause of the AE. Resolution of AEs (with dates) will be documented in the CRF and in the patient's medical record. Reporting of SAEs to the IRB will be done in compliance with the standard operating procedures and policies of the IRB and with applicable regulatory requirements. Documentation will be kept throughout the study and provided to the IRB as needed.

Each international site will require local IRB review. VUMC as the coordinating site will be made aware of adverse events for close record keeping including resolution and outcome of reported events at the local site.

Describe plans for assuring that any action resulting in a temporary or permanent suspension of a federally funded research project is reported to the grant program director responsible for the grant.

N/A

Describe plans for assuring data accuracy and protocol compliance.

Qualified individuals will monitor all aspects of the study according to good clinical practice and standard operating procedures for compliance with Vanderbilt regulations and the IRB. The study PI will allow these monitors direct access to the clinical supplies; dispensing and storage areas; and the clinical files, including original medical records, of the study subjects. The investigator also agrees to assist the monitors if requested.

We will conduct regular review of the study protocol and good clinical practice on a monthly basis as a study team participating in the clinical intervention.

Training of international sites and the VUMC protocol will be provided. Weekly meetings will require review of compliance of international sites.

Is Vanderbilt going to be the Coordinating Center?

[**x**] **Yes**
[ ] No

Protocol and/or case report form development and/or distribution?

[**x**] **Yes**
[ ] No

Sample consent form development and/or distribution?

[**x**] **Yes**
[ ] No

Date of IRB Approval: 12/20/2018
Institutional Review Board
VANDERBILT

Describe the coordinating center's role in reviewing modifications by the collaborating institution of sample consent information related to risks or alternative procedures to assure changes are appropriately justified.

Modifications to the protocol by the collaborating institution will require written appeal and approval by the coordinating center at VUMC. We will provide a sample consent form, but the collaborating institution will require their own local IRB approval.

Critical documents (study) management?

[**x**] **Yes**
[ ] No

Describe the types of documents the coordinating center is responsible for managing.

We will require copies of IRB approved documents from each participating site prior to enrollment of the first subject.

Site selection and training in study procedures?

[**x**] **Yes**
[ ] No

Describe site selection, qualifications, site training and how training will be provided.

Sites were funded and selected through the funding agency the Foundation for Angelman Syndrome Therapeutics in Aunistralia. Sites will require access to all required studies including EEG, ERP, gait tracking. It should be an academic center with access to their own IRBs. Training will be provided remotely through conferences and by providing strict protocol directions. We will continue to meet regularly with the site PIs to address any concerns and protocol deviations.

Assuring informed consent is obtained from each participant enrolled at the participating centers?

[**x**] **Yes**
[ ] No
[ ] N/A

Describe the mechanisms to be employed.

The international sites must provide signed informed consents for each study participant to the VUMC coordinating center prior to the first study visit.

Tracking of serious adverse events and unanticipated problems involving risk to participants or others, reporting to participating centers and regulatory reporting?

[**x**] **Yes**
[ ] No

Date of IRB Approval: 12/20/2018
Institutional Review Board
VANDERBILT

Describe who will be responsible for receiving and reviewing serious adverse events and unanticipated problems involving risk to participants or others reported by the participating centers and how those reports will be disseminated to other participating centers, the coordinating center and participating site IRBs, sponsors, data safety monitoring boards and applicable regulatory agencies.

Participating site IRB will be immediately responsible for the site locally at the time of learning of the adverse event. This should be handled per the local IRB protocol. The local sites will each have data safety monitoring board and will be managed by a local CRO who will have the initial immediate responsibility to ensure safety of the participants in the study.

The VUMC PI will review any serious adverse events and be responsible for immediate discussion with the collaborating center. If there is an identified risk to study participants these concerns will immediately be discussed with all other collaborating centers in a conference call that also includes the directly involved PI. The full committee will review each adverse event and a consensus decision will be made to determine how best to handle the situation.

Will the coordinating center receive/store private, identifiable information about study participants from the participating centers?

[ ] Yes
[**x**] **No**

Will coordinating activities include responsibilities that require contact with participants from the participating centers?

[ ] Yes
[**x**] **No**

Statistical Analysis?

[**x**] **Yes**
[ ] No

Please describe who will perform the statistical analysis and the qualifications of the individual performing the statistical analysis. In addition, describe whether the analysis will involve identifiable samples/information.

Data analysis including statistical analysis of all data (from the coordinating site and the collaborating centers) will be conducted by Dr. Chris Slaughter in the Biostatistics department under the current protocol approved at VUMC. Data will be analyzed in aggregate to increase the power of the study.

Publication or Presentation of Study Results?

[**x**] **Yes**
[ ] No

Date of IRB Approval: 12/20/2018
Institutional Review Board
VANDERBILT

Do you have any other Coordinating Center responsibilities?

[ ] Yes
[**x**] **No**

Please list all sites for which the Coordinating Center is responsible:

2 sites in Australia

Is Vanderbilt also recruitment site?

[ ] Yes
[**x**] **No**

Please select the phase of study.

[ ] Phase I
[ ] Phase I/II combined
[ ] Phase II
[ ] Phase III,
[ ] Phase IV
[**x**] **N/A**

Does this study require registration with clinicaltrials.gov?

[**x**] **Yes**
[ ] No

Subject Population(s)

Is this a study in which you will have interaction with individuals? NOTE: Please check "yes" if you need to report accrual goals for participant engagement.

[x] **Yes**
[ ] No

Accrual Goal: What is your total accrual goal at Vanderbilt?

15

Total number of participants stated in the protocol to be studied at all sites (regardless of PI).

30

Does this study target one gender or specific social/ethnic group(s)?

[ ] Yes
[x] **No**

Is the population being enrolled in this study at high risk for incarceration?

[ ] Yes
[x] **No**

Check all that apply (*Complete the appropriate supplemental information as applicable):

[ ] N/A
[x] **Children/minors***
[ ] Cognitively impaired - comatose/traumatized*
[ ] Pregnant women/fetal tissue/placenta*
[ ] Prisoners*

Place a check in the box beside the category that best describes your proposed research. If the research study includes two pediatric populations (i.e. a healthy control/donor group and a patient/recipient group), identify the appropriate category and provide justification for each group individually.

[ ] (45 CFR 46.404; 21 CFR 50.51) This proposed research poses no greater than minimal risk to children.
[x] **(45 CFR 46.405; 21 CFR 50.52) This proposed research poses greater than minimal risk to children and includes an intervention or procedure that DOES hold out the prospect of a direct benefit for the individual child or a monitoring procedure that is likely to contribute to the child's well-being.**
[ ] (45 CFR 46.406; 21 CFR 50.53) This proposed research poses greater than minimal risk to children and is presented by an intervention or procedure that DOES NOT hold out the prospect of direct benefit for the individual subject, or by a monitoring procedure which is not likely to contribute to the well-being of the subject, BUT is likely to yield generalizable knowledge about the subject's disorder or condition.
[ ] (45 CFR 46.407; 21 CFR 50.54) This proposed research does not meet the requirements of Category 1, 2, or 3 listed above. The IRB will submit the study to the Office of Human Research Protections (OHRP) for review and certification.

Date of IRB Approval: 12/20/2018
Institutional Review Board
VANDERBILT

Please describe how the risk is justified by the anticipated benefit to the child.

The potential benefits of the study to participants include transition to an alternative fuel for the body which may have benefits for refractory seizures (as shown by data on the ketogenic diet and LGIT therapy in AS), cognition, and mobility (as suggested by data from animal models). This may also help families with induction of ketosis on standard diet, or improved or sustained ketosis in those participants on LGIT or ketogenic diets. The risks include the standard risks of venipuncture and the possible side effects which are primarily gastrointestinal discomfort associated with the study formulation. Available data on agents to induce ketosis when used with ketogenic diet suggest low risk of side effects including hypoglycemia.

Please describe how the relation of the anticipated benefit to the risk is at least as favorable to the subjects as that presented by available alternative approaches.

Alternative approaches include induction of ketosis with use of the ketogenic diet and LGIT diets. These diets are very restrictive and individuals have difficulty with compliance due to unpalatable nutrition options to maintain the benefits of ketosis. The goal of the formulation is to garner some of the benefits clearly shown from these approaches especially with respect to seizures without the restriction of diet or to allow for a more liberal diet. This is essential especially in a population of individuals that have unpredictable sensory aversions. This formulation has the same risks as these approaches, which are directly related to the transition of the body to an alternative fuel.

Date of IRB Approval: 12/20/2018 Institutional Review Board VANDERBILT

Please describe how adequate provisions are made for soliciting the assent of the children and the permission (parental/guardian informed consent) of both parents or guardians is sought.

The consent form will initially be presented to the family via telephone once the family has received a copy of the consent sent via FEDEX to their home. The family will be provided the option to sign and return the form or wait to sign the consent when at the initial study visit. No activities related to the study will be performed prior to obtaining a signed consent form. The study team will read the assent script accordingly to the participating child. The study team will always attempt to get both parents signature for the informed consent, if one of the parents cannot come to the visit to sign the informed consent, then the informed consent will be sent by overnight delivery FEDEX with tracking number to the parent that cannot come to the visit and will be reviewed with the parent on the telephone. A preaddressed and pre-paid fedex envelope will be mailed with the informed consent so that the parent can sign the consent form and mail out without any additional expense to the parent. Once the Informed consent is signed by the consenter at Vanderbilt, a copy of the complete consent form will be overnighted to the parent.

The study team will describe the study, including all the requirements for informed consent. The study team will give ample time for questions. The study team will answer all questions posed to it. The study team will ask for a signature from the parent of a child with AS. We will attempt to receive a signed consent from both parents.

We do not expect the participants to possess a cognitive ability greater than that of a 5-year-old child or have developed language skills. Gaining verbal assent from these patients is not practicable. For this reason, it is imperative the parent or guardian have a full understanding of the potential benefits, risks and study requirements explained in the informed consent form prior to providing consent for their child to participate. We will at least attempt to explain the study process, the potential risks, benefits, and study requirements and answer in detail any questions the child may have on a level they can understand. Further, they will be advised that they have the option to not participate and if they choose not to participate their parent or guardian and the study staff will not be upset with them.

In the rare case a potential participant has the ability to understand and communicate their wishes we will seek verbal assent from potential participants younger than 7 years old and written assent from those greater than 7 years old. A copy of the assent script will be signed and dated by the PI or Study Coordinator and stored in the clinic record as documentation assent was given by the potential participant (Appendix E). The assent process will be repeated during each follow-up visit. Assent will only occur in person during study visits.

Please Include any dissenting behaviors that when observed may result in the child being withdrawn from the research.

Dissenting behaviors may include, but are not limited to children refusing to participate in research study procedures, parents not bringing child to scheduled appointments per protocol guidelines. If the safety of the child is in question by parents not adhering to study protocols, or if the child or parent ask to no longer be in study this will be result in conclusion of participation. If safety of the child in anyway appears to be in question this will result in immediate withdrawal from the study.

Date of IRB Approval: 12/20/2018
Institutional Review Board
VANDERBILT

Recruitment

Describe the specific steps to be used to identify and/or contact prospective participants. (If applicable, also describe how you have access to lists of potential participants.)

The study PI focuses her work on AS among other epigenetic disorders. Through patients seen in divisions of genetics and neurology, participants will be recommended for the study. The study coordinator will reach out to these families and gauge potential interest in participation. Additionally, through local patient support and advocacy groups individuals will be provided information regarding the study. Websites and social media groups will advertise the study to the AS rare disease community to identify prospective participants. We may access list of participants from the local support groups if permission is obtained prior to obtaining these lists.

Vanderbilt will only recruit participants for the Vanderbilt study site.

Identify the criteria for inclusion and exclusion and explain the procedures that will be used to determine eligibility. If psychiatric/psychological assessments will be conducted (e.g., depression or suicidal ideation screenings), state who will administer, his/her experience, and how risks will be managed.

Inclusion Criteria:
• Male or female
• 4-11 years of age
• Diagnosed with Angelman Syndrome with prior lab report indicating genetic diagnosis
• Motivated to follow the LGIT or KD for at least the duration of the trial period
• Currently on a LGIT, KD, or standard diet consistently for at least three months
• Written informed consent from patient and/or parent/caregiver
• Willing to drink provided formulations, or to eat them mixed with food
• Daytime toilet trained, or parents willing to use cotton balls in diapers to sample urine
• Willing to comply with protocol examinations, including EEG, ERP, and venipuncture
• Ambulatory, willing to perform gait tracking

Exclusion Criteria:
• Being pregnant or planning pregnancy
• Requiring parenteral nutrition
• Major hepatic or renal dysfunction
• Participation in other clinical intervention studies within 1 month prior to entry of this study
• Allergy to any of the study product ingredients
• Investigator concern around willingness/ability of patient or parent/caregiver to comply with protocol requirements
• Any contraindications for the use of the ketogenic or low carbohydrate diets
• Significantly underweight (Body Mass Index <18.5)
• Unwilling to drink provided formulations, or to consume formulation mixed with food
• Not ambulatory, or unwilling to perform gait tracking
• Not daytime toilet trained, or parents unwilling to use cotton balls in diapers to sample urine
• History of or concern for diabetes

The study PI, team and CSO will review potential candidates based on information obtained from the potential participants medical history including as needed review with the individuals medical team to ensure all the inclusion and exclusion criteria are met.

Date of IRB Approval: 12/20/2018
Institutional Review Board
VANDERBILT

Describe how the selection of participants is equitable in relation to the research purpose and setting.

The purpose of the study is to underscore AS and the safety and tolerability of the formulation in individuals with AS. Selection of participants will be based on standard inclusion and exclusion criteria.

No single ethnic group will be targeted. The study participants and the entire Angelman community will benefit from the study results.

Please identify ALL applicable recruitment methods:

[ ] N/A
[**x**] **Flyers**
[**x**] **Internet**
[**x**] **Letter**
[ ] Departmental Research Boards,
[**x**] **Mass E-mail Solicitation/Research Notifications Email Distribution List**
[ ] Newspaper
[**x**] **Posters**
[ ] ResearchMatch (IRB 090207)
[ ] Radio
[ ] Telephone
[ ] Television
[**x**] **Social Media**
[ ] Other

Will the study provide compensation to research participants?

[**x**] **Yes**
[ ] No

Please specify the method of compensation.

$100

Please include information describing the payment amount and schedule.

The participants will receive a total of $100 in Amazon gift cards. This will be evenly distributed throughout the study and will be given to participants at the completion of the each CRC visit (4 total) in the study.
Each gift card will be in the amount of $25.00 and each one will be given at the completion of each of the 4 visits.

Visit 1: 25.00 gift card will be given to the participant
Visit 2: 25.00 gift card will be given to the participant
Visit 3: 25.00 gift card will be given to the participant
Visit 4: 25.00 gift card will be given to the participant

Are you requesting a waiver for the collection of Social Security numbers?

[ ] Yes
[**x**] **No**

Do you agree to release study information to Vanderbilt-approved list services, web sites or publications? NOTE: Vanderbilt has a variety of list services and publications, such as the Clinical Trials Website. Posting research protocol information on research-related websites and other listing services, allows potential participants to search and find studies related to their condition or interest.

[**x**] **Yes, this information may be released as described in the lay summary.**
[ ] No, do not release information to research-related web sites and other listing services.

Does this study include a certificate of confidentiality or sensitive research information that must be hidden in the medical record?

[ ] Yes
[**x**] **No**

Radiation Procedures and Radioactive Drugs

Does this study involve any radiation ionizing procedures for research?

[ ] Yes
[**x**] **No**

Drugs, Devices, Biologics

Please check all that apply:

[**x**] **N/A**
[ ] Drug(s)/Biologic(s) or Placebo (inactive substance) Used for Research that HAVE an IND
[ ] Drug(s)/Biologic(s) or Placebo (inactive substance) used for Research that DO NOT have an IND [only include drugs that are being used outside of package insert labeling for indication, route of administration, dose, dosing frequency, dosage form, and/or population in which the drug is being used (i.e. children)]
[ ] IBC Review for Live, Recombinant, and/or Attenuated Microorganisms for Vaccination, Gene Transfer or Botox
[ ] Device(s) Used for Research (devices may also include computer software, in vitro diagnostics, etc.)

Date of IRB Approval: 12/20/2018
Institutional Review Board
VANDERBILT

PHI/Consent

Please indicate what you plan to do with regard to consent (check all that apply):

[**x**] **Consent**
[ ] Waiver of Consent
[ ] Consent was obtained in another study
[ ] Consenting not required (e.g., exempt project, non-human project)

Please describe the specific steps for obtaining informed consent and the procedures that will be utilized to protect the privacy of individuals.

The study team (either Study coordinators (either a Registered Nurse or Nurse Practitioner) or Principal Investigator (Medical Doctor) will describe the study, including all the requirements for informed consent on an initial phone call to the prospective participant's family. The phone call will not take place until the family has confirmed receipt of the consent form. The study team will give ample time for questions. The study team will answer all questions posed to it. The study team will ask for a signature from both parents of a child with AS. Every attempt will be made to have signatures from both parents, although if one parent is not available a single signature will be considered sufficient for participation. The study team will also obtain assent or read the assent script accordingly to the child.

The study team will always attempt to get both parents signature for the informed consent. Informed consent will be sent by overnight delivery FEDEX with tracking number to the parent/caregiver and will be reviewed with the parent on the telephone. A pre-addressed and pre-paid fedex envelope will be mailed with the informed consent so that the parent can sign the consent form and mail out without any additional expense to the parent. Once the Informed consent is signed by the consenter, a copy of the complete Consent form will be provided to the parent.

Does the person obtaining consent have an existing relationship with the participant(s)?

[**x**] **Yes**
[ ] No

Please indicate/describe the relationship(s) and how you will protect against undue influence or coercion.

In order to avoid influence, the study PI or other physicians making referral to the study will only obtain consent for additional information be provided to the family by the study team at a later date. A member of the study team will then reach out to the family independent of the appointment to describe the study and offer an appointment to give more information. The family will be given ample time to consider participation and to ask questions. Every effort will be made to obtain consent from each parent or guardian (if possible).

Please describe any waiting periods between informing potential participants of the research and obtaining consent, if applicable.

The period will be approximately one month between informing participants of the research, a member of the study team reaching out and then scheduling an in-person appointment to obtain consent.

Will surrogate consent be requested?

[ ] Yes
[**x**] **No**

Date of IRB Approval: 12/20/2018
Institutional Review Board
VANDERBILT

How will non-English speaking participants be consented?

[ ] A translated written informed consent document in a language understandable to the participant. This should be an accurate translation of the full informed consent document (consider having a translator present during the consenting process should the participant have any questions).
[ ] Orally, using a qualified translator to translate the English informed consent document to the participant, and a translated short form in a language understandable to the participant. (See "Documentation of Informed Consent" at IRB Policy IV.B for details).
[**x**] **Enrolling only English speaking participants.**

Please provide a justification for only enrolling English speaking participants.

We will initially enroll English-speaking patients due to the complexity of the studies to be completed and the lack of instructions available in other languages. We will aim to over time prepare instructions in Spanish to broaden the ethnic diversity. However, prior to this time we will aim to first begin the study by enrolling English-speaking patients only. An amendment will be submitted to diversity the study once ample time has allowed us to prepare the necessary instructions.

Please describe the plan to protect the identifiers from improper use and disclosure.

Patients will be assigned a unique numeric identifier. This number will be used to identify the subject. The key will be held securely by the study team and will not be transmitted. Electronic case report forms (eCRFs) will be provided for each subject using a validated web-based application. All users will be trained on the eCRF application prior to being granted system access. Data will be stored separately with PHI in a password protected database only accessible to key study personnel.

Will Protected Health Information (PHI) be accessed (used) in the course of screening/recruiting for this research?

[**x**] **Yes**
[ ] No

Does this research use or disclose Protected Health Information (PHI)?

[**x**] **Yes**
[ ] No

Please indicate the source of the PHI to be collected.

PHI will be collected from medical records, specimens and patients and their families

Please indicate when PHI will no longer be accessed.

Upon completion of the study including analysis of study results.

Please describe the plan to destroy the identifiers at the earliest opportunity consistent with the conduct of the research, unless there is a health or research justification for retaining the identifiers or such retention is otherwise required by law.

All study documents must be kept for up to 3 years but once the study is closed for 6 months to 1 year then documents will be sent to an outside documentation storage location called Iron Mountain

Conflict of Interest Disclosure

Is there a potential conflict of interest for the Principal Investigator or key personnel? • The PI is responsible for assuring that no arrangement has been entered into where the value of the ownership interests will be affected by the outcome of the research and no arrangement has been entered into where the amount of compensation will be affected by the outcome of the research. • Assessment should include anyone listed as Principal Investigator, or other research personnel on page 1 of this application. Please note that ownership described below apply to the aggregate ownership of an individual investigator, his/her spouse, domestic partner and dependent children). Do not consider the combined ownership of all investigators.

[ ] Yes
[**x**] **No**
