# Supplementary material for: Evaluation of the safety and tolerability of a nutritional Formulation in patients with ANgelman Syndrome (FANS): study protocol for a randomized controlled trial
Source: Trials. 2020 Jan 9;21:60. doi: 10.1186/s13063-019-3996-x (PMC6953273; doi:10.1186/s13063-019-3996-x)
Supplement: Supplementary file 4 — Additional file 4. Additional protocol details. [file 13063_2019_3996_MOESM4_ESM.docx]

**Additional File 4: Additional Protocol Details**

**Reporting of Adverse Events or Unanticipated Problems Involving Risk to Participants or Others**

**Definitions:** The following definitions were adapted from the Food and Drug Administration’s Rule 21 CFR Part 312.32, IND Safety Reports for Clinical Trials.

1. Unanticipated Problems Involving Risk to Participants or Others

Any incident, experience, or outcome that meets all of the following criteria:

- Unexpected in nature, severity, or frequency (i.e. not described in study-related documents such as the IRB-approved protocol or consent form, the investigators brochure, etc.)
- Related or possibly related to participation in the research (i.e. possibly related means there is a reasonable possibility that the incident experience, or outcome may have been caused by the procedures involved in the research)
- Suggests that the research places participants or others at greater risk of harm (including physical, psychological, economic, or social harm).

1. Adverse Event - An adverse event (AE) is any symptom, sign, illness or experience that develops or worsens in severity during the course of the study. Intercurrent illnesses or injuries should be regarded as adverse events. Abnormal results of diagnostic procedures are considered to be adverse events if the abnormality:

- results in study withdrawal
- is associated with a serious adverse event
- is associated with clinical signs or symptoms
- leads to additional treatment or to further diagnostic tests
- is considered by the investigator to be of clinical significance

1. Serious Adverse Event

Adverse events are classified as serious or non-serious. A serious adverse event is any AE that is:

- fatal
- life-threatening
- requires or prolongs hospital stay
- results in persistent or significant disability or incapacity
- a congenital anomaly or birth defect
- an important medical event

Important medical events are those that may not be immediately life threatening, but are clearly of major clinical significance. They may jeopardize the participant, and may require intervention to prevent one of the other serious outcomes noted above. All adverse events that do not meet any of the criteria for serious should be regarded as non-serious adverse events.

1. Adverse Event Reporting Period

The study period during which adverse events must be reported is normally defined as the period from the initiation of any study procedures to the end of the study treatment follow-up. For this study, the study treatment follow-up is defined as 30 days following the last administration of study treatment.

1. Preexisting Condition

A preexisting condition is one that is present at the start of the study. A preexisting condition should be recorded as an adverse event if the frequency, intensity, or the character of the condition worsens during the study period.

1. General Physical Examination Findings

At screening, any clinically significant abnormality should be recorded as a preexisting condition. At the end of the study, any new clinically significant findings/abnormalities that meet the definition of an adverse event must also be recorded and documented as an adverse event.

1. Post-study Adverse Event

All unresolved adverse events should be followed by the investigator until the events are resolved, the participant is lost to follow-up, or the adverse event is otherwise explained. At the last scheduled visit, the investigator should instruct the parent or guardian of the participant to report any subsequent event(s) that the participant to the participant’s personal physician, believes might reasonably be related to participation in this study.

1. Hospitalization, Prolonged Hospitalization or Surgery

Any adverse event that results in hospitalization or prolonged hospitalization should be documented and reported as a serious adverse event unless specifically instructed otherwise in this protocol. Any condition responsible for surgery should be documented as an adverse event if the condition meets the criteria for an adverse event. Neither the condition, hospitalization, prolonged hospitalization, nor surgery are reported as an adverse event in the following circumstances:

- Hospitalization or prolonged hospitalization for diagnostic or elective surgical procedures for a preexisting condition. Surgery should not be reported as an outcome of an adverse event if the purpose of the surgery was elective or diagnostic and the outcome was uneventful.
- Hospitalization or prolonged hospitalization required to allow outcome measurement for the study.
- Hospitalization or prolonged hospitalization for therapy of the target disease of the study, unless it is a worsening or increase in frequency of hospital admissions as judged by the clinical investigator.

**Recording of Adverse Events**: The study PI or coordinator will record all adverse events in the form of a report including a description of the event, the category of the event (according to the above definitions) and a preliminary assessment of the relationship between the AE and the study treatment. The report will be review by the study investigator(s) to determine whether it is safe for the subject to continue the study and maintained as part of the permanent study records.

**Reporting of Serious Adverse Events and Unanticipated Problems:** A written report will be filed with the FDA by the study PI or coordinator within 15 calendar days of learning of an adverse event that is related to the study formulation. The FDA will be notified of all fatal or life-threating adverse events no later than 7 calendar days after notification of the event. The Study PI and/or coordinator will be responsible for reporting any unanticipated problems involving risk to participants or others (UPIRHSO) and serious adverse events to the Vanderbilt University Institutional Review Board as soon as becoming aware of the event.

**Medical Monitoring:** As part of the protocol closeout visit (at 4 weeks post administration), a survey of the participants parent or guardian will ask specific questions designed to elicit known side effects of consuming the study formulation. Further, the parent or guardian will be instructed to notify the study coordinator promptly of all major medical conditions that may arise or observed changes in behavior during the study course. Examples of circumstances include: major illness, surgical procedures, hospitalizations, changes in sleep patterns and behavior that is unusual for the child. If the participants behavior is considered by the study PI or coordinator to be very unusual, bizarre, or dangerous the parent or guardian will be advised to have the child evaluated by the study neurologist (if local) or the child’s primary care physician.

**Data Safety Monitoring Plan**: The individuals responsible for data safety monitoring on a day to day basis will be the principal investigator, study neurologist and study coordinator. The Data Safety Monitoring Board will meet periodically (specific dates to be determined by the DSMB chairperson) to review the study data and any adverse events. The DSMB responsibilities are described below.

Quality control will include regular data verification and protocol compliance checks by the study coordinator. The study coordinator will complete monthly reports detailing the study progress and subject status, any adverse events, and any protocol deviations. Protocol adherence will be monitored by the study coordinator. The reports will be reviewed by the study principal investigator regularly. The reports will be made available to the DSMB so they may be reviewed at the earliest DSMB meeting.

Throughout the study, the study coordinator will monitor the participants for adverse events in real time and report any occurrence to the study principal investigator and study neurologist. Events determined by the principal investigator (PI) and neurologist to be unanticipated problems involving risk to participants or others (UPIRHSO) as defined in this study protocol will be reported by the PI to the IRB as soon as becoming aware of the event. Adverse events determined by the PI and study neurologist not to be UPIRHSO’s will be reported to the IRB at the time of continuing review. If an event is determined by the PI and study neurologist to be a serious adverse event, as defined in this study protocol, the study will be temporarily halted until such time as it can be determined by the study PI and study neurologist if the event was associated with the study formulation. The study PI will report a serious adverse event to the Vanderbilt University IRB immediately after becoming aware of the event.

All study staff members will be informed of the UPIRHSO. If any protocol changes are needed, the PI or study coordinator will submit a modification request to the IRB. Protocol changes will not be implemented prior to IRB approval unless necessary to eliminate apparent immediate hazards to the research participants. In such as case, the IRB will be promptly informed of the change following implementation within 10 working days.

Statistical review of the study data will be conducted by the DSMB biostatistician at time points determined by the DSMB chairperson.

**Data Management**

Each participant will be assigned a unique study number by the study PI or coordinator to protect the participant’s personal health information (PHI). Personal information will only be used by the study coordinator in the clinical setting. A master database containing participants names and unique study number will be stored as a secured excel file on the study PI’s computer and a printed copy in a locked file cabinet in the PI’s office. All data collected from the study participants will be de-identified prior to leaving the clinical setting by entering specific data points into an electronic data collection program according to study number. This process will protect the participant’s personally identifiable information.

Data management will be the responsibility of the study coordinator. He/she will maintain the electronic study database, study worksheets, and informed consent. Although any document leaving the clinic setting is to be de-identified, all study data will be stored securely in electronic format on the Vanderbilt University secure server. Despite deidentification, only HIPPA trained study personal will have access to the study data base.

Source documents will include clinical surveys, ERP and EEG tracings, gait analysis pressure recordings, data captured in apps on the provided tablet, and parent questionnaires.

It will be the investigator’s responsibility to maintain essential study documents for a minimum of 3 years from the date of the last participants close out clinical visit. The investigator will permit study-related monitoring, audits, and inspections by the IRB, government regulatory bodies, and University compliance and quality assurance groups of all study related documents (e.g. source documents, regulatory documents, data collection instruments, study data etc.). The investigator will ensure the capability for inspections of applicable study-related facilities (e.g. clinic, diagnostic laboratory, etc.). Participation as an investigator in this study implies acceptance of potential inspection by government regulatory authorities and applicable University compliance and quality assurance offices.

**Privacy/Confidentiality Issues**

This study will be performed according to all Vanderbilt University privacy policies as well as federal, state and local regulation. In accordance with HIPPA regulations all “individually identifiable health information” will be removed (de-identified) from any source documents before leaving the clinical site. Participants enrolled in this study will be assigned a study number which will be used to identify a particular data source. A master list will be made containing patient name, date of birth and study number and stored securely in a lock filing cabinet in the investigators office. The investigators office is located in a secured building requiring badge access and will be locked at all times when the investigator is not present. Deidentification of study data will be achieved by reviewing source documents while in the clinical setting and transferring specific data points into a secure data collection software program according to a participants study number. Data will be stored on a Vanderbilt University secure server. Source documents will be stored securely according to the Vanderbilt University standard operating procedures.

**Publication Plan**

Statistically significant results will be published in peer reviewed journals and presented at professional meetings. Further it is our intention to share the results of this study with any other interested clinicians and scientist working to find a cure for this or another syndrome. It is our intention to make the information gathered from this study available to all, potentially through the use of patient registries.
